# Nephrotoxicity of methadone: a systematic review

**DOI:** 10.1186/s40064-016-3757-1

**Published:** 2016-12-09

**Authors:** Samira Alinejad, Kazem Ghaemi, Mohammad Abdollahi, Omid Mehrpour

**Affiliations:** 1 Medical Toxicology and Drug Abuse Research Center (MTDRC), Birjand University of Medical Sciences, Moallem Avenue, Birjand, 9713643138 Iran; 2 Atherosclerosis and Coronary Artery Research Centre, Birjand University of Medical Sciences, Birjand, Iran; 3 Department of Neurosurgery, Birjand University of Medical Science, Birjand, Iran; 4 Toxicology and Diseases Group, Pharmaceutical Sciences Research Center, Tehran University of Medical Sciences, Tehran, Iran

**Keywords:** Methadone, Kidney, Renal, Toxicity, Rhabdomyolysis

## Abstract

**Background:**

Methadone is commonly administered for chronic pain relief and treatment of opioid dependence. Concurrent with its increased consumption, toxicities and fatalities have increased. One of the adverse effects of opioid analgesics, including methadone, is that of nephrotoxicity. Opioids can have an effect on renal function through several different mechanisms.

**Methods:**

We searched common bibliographical databases for the terms methadone, toxicity, poisoning, kidney, renal, and nephrotoxicity and summarize our findings in this review.

**Results:**

Methadone can have both direct and indirect effects on the kidney. These effects include rhabdomyolysis (leading to acute kidney injury), volumetric changes, renal lipidosis and amyloidosis, kidney growth during pregnancy, and kidney transplant rejection.

**Conclusion:**

Improved understanding of the effects of methadone on kidney function can promote safer and more confident use of the drug.

## Background

Opioid dependence as a chronic condition is associated with morbidity and mortality (Bell et al. 2009; Karrari et al. 2013). In Iran there is a high prevalence of opioid abuse due to its geographic location in the Middle East and having a young population (Hassan Ziaaddini et al. 2011; Mehrpour and Sezavar 2012; Karrari et al. 2012).

Methadone is a synthetic mu-opioid receptor agonist widely used to treat opioid dependence (Alinejad et al. 2015; Grissinger 2011). It was first developed in Germany in 1937 and introduced to the US in 1947 by Eli Lilly and Company under the trade name of Dolophine (Lipman 2008; Trafton and Ramani 2009). Since the mid-1960s, it has been used for the treatment of opioid dependence (Amiri-Aref et al. 2013; Nazari 2007). It is now on WHO (World Health Organization) list of essential medicines (Pilgrim et al. 2013). More than 30,000 and 250,000 patients have been treated in methadone maintenance programs in Australia and America, respectively (Pilgrim et al. 2013; Thanavaro and Thanavaro 2011). In Iran, there are currently more than 1500 methadone centers (Aghabiklooei et al. 2014).

Methadone has pharmacokinetic and pharmacodynamic properties that make it attractive for treating patients with opioid dependence (Fahey et al. 2003; Strain 2002). Nevertheless, similar to other opioid medications, it has a potential for abuse and can either cause death directly or contribute to fatality indirectly (Amiri-Aref et al. 2013; Van Den Broecke et al. 2012). Methadone-related reports of deaths have been accumulating over the past 40 years (Modesto-Lowe et al. 2010).

One of the potential adverse effects of opioid analgesics including methadone is nephrotoxicity (Atici et al. 2005; Lentine et al. 2015). Opioids influence renal function through various mechanisms and can cause or exacerbate a wide range of kidney diseases (Crowe et al. 2000; Mercadante and Arcuri 2004). The safe and effective administration of opioids can be an important concern in patients with impaired renal function (Niscola et al. 2010).

The issue of patients’ individual responses to pain intensity, tolerance, and experience of adverse effects necessitates an understanding of the relationship between opioids and renal function (Melilli et al. 2014; Mercadante and Arcuri 2004; Murtagh et al. 2007). In this article we review methadone’s effects on the kidney.

## Methods

**Fig. 1:**
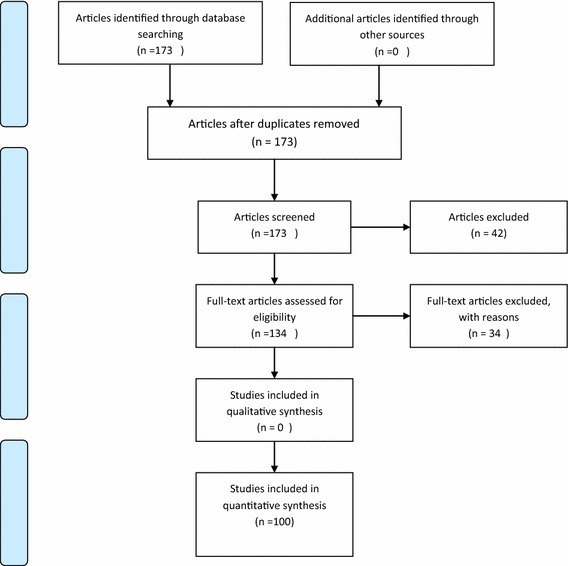
PRISMA 2009 flow diagram

A search was done on the terms methadone, toxicity, poisoning, kidney, renal, and nephrotoxicity in TUMS digital library, PubMed, Scopus, EmBase, and Google Scholar bibliographical databases. This review included the articles published between 2000 and 2015 though some very relevant articles published before 2000 were also taken into account. We excluded articles reporting animal studies and articles concerning the use of methadone in renal disease patients (Fig. 1).

This article is a literature review and does not contain data from any intervention studies we performed. A formal consent was not required for this type of the study.

## Methadone pharmacokinetics

Methadone’s chemical structure is different from that of a typical opioid by having the form of an open-chain amine. Having a strong affinity with a µ-opioid receptor, it is classified as a diphenyl-heptane derivative (Trescot et al. 2008).

Methadone has an oral bioavailability of 70 to 90% and reaches peak plasma concentrations within 2–4 h after being ingested, while its analgesic effect appears almost 15 min after a subcutaneous injection. It has a long but variable plasma half-life of 15–55 h with a mean of approximately 24 h. Plasma protein binding in plasma is 60–90%. Methadone is widely distributed among tissues, with an apparent volume of distribution (Vd) of 2–6 L/kg. It is metabolized in the liver, undergoing N-methylation and cyclization to be eliminated in an unconjugated form (Brown et al. 2004; Ferrari et al. 2004). Cytochrome P450 (CYP) isozymes 3A4, 2D6, and to a lesser extent 1A2 are involved in metabolism. CYP3A4 enzyme activity may vary up to 30-fold between individuals. Moreover, a small percent of the population entirely lacks CYP2D6 enzyme (Gevirtz 2007; Kharasch et al. 2004; Mehrpour 2013; Shinderman et al. 2003).

Up to 40% of a dose of methadone is eliminated by the kidney, depending on the urine pH. Renal drug clearance is significantly enhanced at a urine pH below 6 (Bernard et al. 2007).

Methadone is administered via oral, intravenous, rectal, subcutaneous, sublingual, and intrathecal routes (Prommer 2010). In Iran and Australia, the main clinical replacement for opioid dependence is methadone syrup (Aghabiklooei et al. 2014; Pilgrim et al. 2013).

Low, intermediate, and high doses of methadone have been defined as doses <50, 50–100, and >100 mg/day, respectively. The dose for methadone maintenance varies between 30 and 125 mg/day (Strain 2002).

## How methadone affects the kidney

Nephrotoxicity can occur as a direct or indirect result of exposure to drugs (Asangansi et al. 2005; Ashley 2004; Dhodi et al. 2014; Kimmel et al. 2001; Orth 2002). Nearly 20% of acute kidney injury (AKI) cases in the community are caused by drugs. Nephrotoxicity induced by drugs among older adults may reach as high as 66% (Bellomo 2006; Kohli et al. 2000; Nash et al. 2002; Naughton 2008).

Addiction to opioids can lead to kidney injury in various ways, such as acute glomerulonephritis caused by blood borne infection from contaminated needles and AKI caused by non-traumatic rhabdomyolysis (Darke et al. 2006). AKI induced by methadone consumption usually results from rhabdomyolysis (Launay-Vacher et al. 2005).

### Rhabdomyolysis and AKI (Table 1)

Rhabdomyolysis is a pathological and life-threatening disease caused by the release of toxic intracellular constituents into the circulatory system following damage to the integrity of the cell membranes of skeletal muscles. Drugs, toxins, trauma, infections, ischemia, and metabolic disorders are the main causes (Allison and Bedsole 2003; Beetham 2000; Chatzizisis et al. 2008; Giannoglou et al. 2007; Holt and Moore 2001; Melli et al. 2005; Moratalla et al. 2008; Tóth and Varga 2009; Warren et al. 2002; Syed et al. 2009). Most important non-traumatic causes include the use of illicit drugs, convulsive seizure, and excessive muscular strain (Welte et al. 2004). It is estimated that exposure to toxic agents and medications are responsible for up to 80% of rhabdomyolysis cases in adults (Talaie et al. 2007). In 1971, Richter et al. described it to be caused by illicit drugs for the first time (Welte et al. 2004). Another study in 1985, described 3 patients who developed rhabdomyolysis following opiate toxicity (Blain et al. 1985). In two later studies, opiate toxicity was found to be the most common cause of rhabdomyolysis (Talaie et al. 2007, 2008). In addition, rhabdomyolysis can be induced by methadone abuse (Criner et al. 2002; Iranmanesh 2015; Gramenz et al. 2010; Nanji and Douglas Filipenko 1983; Aghabiklooei et al. 2014; Eizadi-Mood et al. 2015).

**Table 1:** Case reports about methadone induced rhabdomyolysis and AKI

Author	Age/gender	Methadone dose	Primary signs and symptoms	Lab parameters	Outcome
Hojs and Sinkovic (1992)	28/Male	30 mg IV	Comatose, cyanotic congenital heart disease, and shallow breathing, anuria	Urea: 124 mg/dl Creatinine: 2.18 mg/dl Myoglobin-positive urine. CK: 12,000 U/L	Discharged in good condition
Chakera (2008)	30/Male	120 mg	Unconsciousness	CK: “increased” Metabolic acidosis, anuria.	After 7 sessions of dialysis, renal function started to recover.
Hsu et al. (2009)	33/Male	150 mg/day (heroin user)	Unconsciousness	CK: 17,680 U/L, Cr: 2.9 mg/dl myoglobin: >4000	Cerebral ischemic infarction
Nanji and Douglas Filipenko (1983)	31/Male	“Large amount” of methadone	Respiratory distress, but awake	BUN: 24 mg/dl, Creatinine: 2.2 mg/dl CK: 7500 U/L.	Recovered
Gramenz et al. (2010)	Not mentioned	10-mg methadone tablets dissolved in water and injected into femoral artery	Severe pain and leg cyanosis. No unconsciousness	CK: 4208 U/L.	Responded to medical therapy
Valga-Amado et al. 2012	41/Male		Muscle weakness and widespread myalgia, Reduced consciousness	Creatinine: 2.88 mg/dl; CK: 86,000 IU/l.	Recovered
Mittal et al.	38/Male	Methadone and diazepam ingestion	Unconsciousness methadone-induced delayed posthypoxic encephalopathy	CK: 3339 IU/l BUN: 13 mg/dl Creatinine: 3.2 mg/dl (diagnosis of AKI was made).	Treated successfully with combination of steroids and antioxidants
Weston et al.	27/Male	Not mentioned	Unconsciousness	CK: 31,500 u/l Urine osmolality: 292 mosmol/l (diagnosis of AKI was made)	Full recovery
David et al. 1	24/Male	40 mg IV	Comatose, Shallow breathing, pulmonary edema	BUN: 62 mg/dl Creatinine: 3.9 mg/dl. CK: 6750 IU/l	Discharged in good condition
David et al. 2	25/Male	50 mg /IV	Apneic, comatose, pulmonary edema	BUN: 132 mg/dl Creatinine: 5.2 mg/dl CK: 190 IU /L	Died

Opioid intoxication leads to rhabdomyolysis through several mechanisms, such as producing prolonged immobility, direct toxic effects from narcotics, allergic reactions to narcotics, muscle tremor to spasm, and sustained hypoxemia (Welte et al. 2004). The direct manner in which methadone may leads to rhabdomyolysis is by means of an increase in the muscular demand for oxygen that augments muscle ischemia (Valga-Amado et al. 2012). Other factors may cause rhabdomyolysis in methadone users: in a case reported by Gramenz et al. a patient injected 10 mg methadone tablets into the femoral artery, which caused limb ischemia without the presence of unconsciousness. The author stated that methadone tablets contain microcrystals of cellulose, which had led to gangrene in the limb (Gramenz et al. 2010).

Rhabdomyolysis may present as asymptomatic elevation of the creatine phosphokinase (CK) serum level, or AKI with steadily rising serum creatinine concentrations (Lindner and Zierz 2003). Other common clinical manifestations include reddish brown urine due to myoglobinuria, muscle weakness, fatigue, pain, and cramps (David 2000; Guis et al. 2005).

The most worrisome complication of rhabdomyolysis is AKI (Deighan et al. 2000; Valga-Amado et al. 2012). Furthermore, in a study on 114 patients with acute poisoning and rhabdomyolysis, AKI was the main complication (Mousavi et al. 2015). Baywaters and Beal first described rhabdomyolysis-induced AKI in 1941 (Chatzizisis et al. 2008). Up to 5–9% of all AKI cases are thought to be caused by rhabdomyolysis and 10–40% of patients with rhabdomyolysis have been reported to develop AKI (Vale 2007). AKI induced by rhabdomyolysis is mainly caused by disrupted muscle cells that release myoglobin. Renal vasoconstriction, ischaemia, formation of myoglobin casts in the distal convoluted tubules, and myoglobin’s direct cytotoxic effect on the epithelial cells of the proximal convoluted tubules are among the purported mechanisms of AKI. Myoglobin’s nephrotoxic action is intensified in the presence of an acidic urine pH caused by metabolic acidosis and hypovolemia (Richards 2000).

In most cases, rhabdomyolysis and AKI occur secondary to a preceding condition such as coma or prolonged immobilization (Corkery et al. 2004). Some examples of relevant studies are presented in Table 1 (Chakera 2008; Hojs and Sinkovič 1992; Hsu et al. 2009; Mittal et al. 2010; Schouwenberg and Deinum 2003; Valga-Amado et al. 2012; Weston et al. 1986).

A presumptive diagnosis of rhabdomyolysis is based on the detection of increases in the activity of creatine kinase (CK) (at least 5 times that of the normal level) in the plasma, or the presence of myoglobin in the urine (Vale 2007).

Reddish brown urine, muscular pain, and weakness are typical symptoms (Galley 2000; Warren et al. 2002). In 50% of patients, the central muscle groups like thighs and shoulders are mainly involved in pain; yet, no muscular symptoms have been reported in over half of the cases. A major diagnostic element is urine reddish-brown color caused by myoglobinuria, which has been observed in almost half of the patients, but the diagnosis cannot be excluded in its absence. Clinical examinations have shown that the skin color changes in response to compression necrosis and swollen and sensitive muscles may appear during palpation (Criddle 2003; Giannoglou et al. 2007).

The diagnosis of rhabdomyolysis, especially during its early phase, might be difficult. Localization of the affected muscle and its differentiation from unaffected muscle groups may be accomplished through radiological techniques. Magnetic Resonance Imaging (MRI) is more sensitive than Computed Tomography (CT) or ultrasound techniques when detecting abnormal muscles. Signal intensities are increased and decreased on T2- and T1-weighted spin-echo images by the affected muscles, respectively. Moratalla et al. (2008) reported a study on an unconscious 31-year old man after methadone consumption. T2- and T1-weighted images of MRI scan had represented diffuse high and low signal intensities in the right upper extremity muscles, respectively, but the latter lacked any intramuscular hemorrhage signs (Moratalla et al. 2008).

### Volumetric changes in the kidney

Opioids may decrease renal function by reducing glomerular filtration (GFR). Reduced systemic blood pressure caused by µ-opioids may cause increased antidiuretic hormone (ADH) secretion and increased central sympathetic outflow, leading to decreased renal perfusion (Shahramian et al. 2009). Also, drugs such as methadone can cause pulmonary edema, and kidneys are very sensitive to hypoxia and respond to it via the infiltration of inflammatory cells (Shahramian et al. 2004, 2009). In an experimental study conducted by Shahramian et al. (2009) on rats, a relative enhancement of kidney volume was found in the methadone group. In another study performed by Jhaveri et al. an acute kidney injury and acid-base disorder following a reduction of haemodynamics and volume depletion occurred after the consumption of 90 mg of methadone (Jhaveri and Webber 2008) (Table 1).

### Renal lipidosis

Renal lipid storage is a phenomenon found in various diseases, and may involve the glomerulus or tubules. Methadone intake and narcotic or intravenous (IV) drug abuse have been associated with this renal pathology. Porubsky et al. described three patients enrolled in a methadone substitution program having renal lipidosis associated with a positive history of narcotic abuse. All three had proteinuria and impaired renal function (Porubsky et al. 2014).

### Renal amyloidosis

Secondary amyloidosis has been described as a cause of renal disease in chronic injection drug users (Buettner et al. 2014; Connolly et al. 2006; Crowe et al. 2000; Jung et al. 2012). Repeated bacterial infections, especially those related to skin and soft tissues, have been associated with renal AA-amyloidosis caused by intravenous intake of methadone (Jung et al. 2012; Miranda et al. 2007; Newey et al. 2007). Nephritic syndrome, proteinuria, and even renal failure are responsible for the clinical manifestations of amyloidosis (Gillmore et al. 2001).

### Kidney growth retardation

Methadone is often given to pregnant women who are in opioid-dependence treatment programs (Wright and Walker 2007). Perinatal methadone has an effect on the kidney as well as body and heart weights, suggesting a fairly generalized action of the drug on organ development. Renal growth and noradrenergic development are affected by methadone (Grignolo et al. 1982). A later study conducted by Ganapathy (2011) in humans supported the idea that the placenta and the developing fetus are influenced by the drugs. It was shown that drug abuse leads to the inhibition of serotonin, neurepinephrine, and dopamine, which, in turn, can enhance the levels of these monoamines within the placenta inter villous space. This would result in vasoconstriction, uterine contraction, compromised placental function, and growth retardation (Ganapathy 2011).

### Kidney transplant rejection

Renal transplant is an important treatment for patients with end-stage renal disease (ESRD) (Monsalve et al. 2011). Adverse clinical outcomes may occur before the kidney transplant procedure in patients with a history of chronic opioid use including methadone (Barrantes et al. 2013).

Lentine et al. conducted a study on 9047 selected kidney recipients with 7 years of opioid use before transplant procedure, who were normalized to morphine equivalents such as methadone. The 3-year graft survival percentage was 84% in the recipients of liver with a history of narcotic use compared to 92% of those not having used narcotics. It was concluded that using high doses of opioid before kidney transplant increases the risks of death and graft loss after transplantation (Lentine et al. 2015).

## Treatment

### Management of rhabdomyolysis (Fig. 2)

Kidney damage can be minimized by early detection and treatment of rhabdomyolysis (Richards 2000). Treatment of the underlying cause of rhabdomyolysis and metabolic acidosis, prevention of AKI, early correction of electrolyte disorders, and management of other complications are the most important therapeutic interventions used in rhabdomyolysis (Chatzizisis et al. 2008). Almost all patients suffering from rhabdomyolysis are hypovolemic, a factor that greatly predisposes them to renal failure because of reduced urine flow and aciduria (Curry et al. 1989). Intravascular volume expansion can enhance renal blood flow, improve glomerular filtration, and increase urine flow, reducing the toxic effects of myoglobin on the tubules. Most clinicians administer intravenous NaCl 0.9% (Holt and Moore 2001). A urinary catheter is recommended to be able to properly monitor hourly urinary output since treatment with a goal of 200–300 ml/h of urine excretion. Excessive administration of fluids can lead to non-cardiac pulmonary oedema, especially those with AKI (Esson and Schrier 2002). Urine alkalization may reduce myoglobin toxicity, and some clinicians use intravenous sodium bicarbonate (NaHCO3) administration (Warren et al. 2002). Serum creatinine and blood urea levels should be frequently checked (Criner et al. 2002). Haemodialysis may be necessary in methadone-induced rhabdomyolysis patients with worsening kidney function (Chaudhari et al. 2015).

**Fig. 2:**
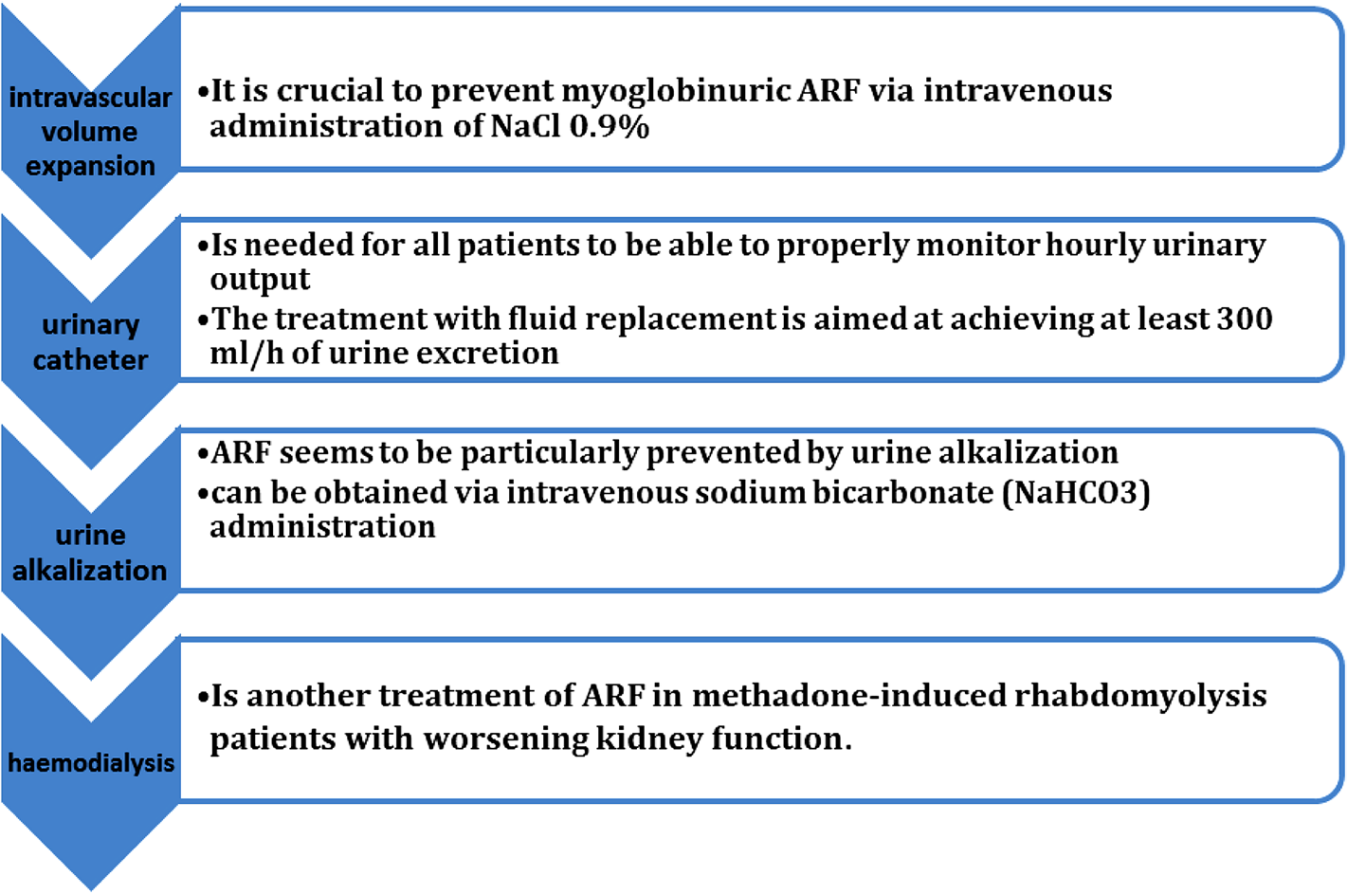
Management of rhabdomyolysis. Treatments of the main cause of rhabdomyolysis and metabolic acidosis, AKI prevention, early modification of electrolyte disorders, treatment, and management of other complications are the most important therapeutic interventions used in rhabdomyolysis

### Discussion

Renal failure is an important cause of mortality and morbidity related to methadone toxicity (Corkery et al. 2004; Aghabiklooei et al. 2014), and rhabdomyolysis is responsible for most cases of AKI following methadone abuse. One of the debates on this topic is the unclear mechanism involved in methadone-induced rhabdomyolysis and AKI (Hojs and Sinkovič 1992). Methadone may induce rhabdomyolysis indirectly, for example as a result of coma leading to compression necrosis of a dependent extremity or respiratory depression causing hypoxemia. For instance, according to the studies conducted by Mittal et al. Hojs et al. Hsu et al. and Valga-Amado et al. unconsciousness causes increased muscular demand for oxygen, as well as secondary ischemia, which may cause rhabdomyolysis (Hojs and Sinkovič 1992; Hsu et al. 2009; Mittal et al. 2010; Valga-Amado et al. 2012) (Table 1). Another explanation for an indirect cause of methadone-induced rhabdomyolysis is pulmonary edema and resultant hypoxia, which also leads to increased muscular demand for oxygen. In the studies conducted by Tóth et al. and Hsu et al. hypoventilation is mentioned as one of the causes of rhabdomyolysis (Hsu et al. 2009; Tóth and Varga 2009) (Table 1). On the other hand, methadone may cause muscle injury as a result of direct toxicity. In a case reported by Gramenz et al. the patient presented with rhabdomyolysis after injection of 10 mg methadone tablets into the femoral artery, which caused limb ischemia without the presence of unconsciousness. The author stated that methadone tablets contain microcrystals of cellulose, which had led to gangrene in the limb (Gramenz et al. 2010). Also the same conclusions were reported in the studies of Nanji and Douglas Filipenko (1983) and Fraser et al. (1971). These studies suggest that methadone itself could be a trigger for the induction of rhabdomyolysis.

Methadone toxicity outcomes can depend on us factors like age, gender, history of suicide attempts and psychological disorders, and the time interval between consumption of the drug and obtaining medical care (Eizadi-Mood et al. 2015). All of the aforementioned cases, except Tóth and Varga (2009) involved young males; also, as described in the original articles, men more frequently present with acute methadone toxicity. The proposed explanation for these findings is that men, especially while young, have a higher tendency to consume illicit drugs. It has been assumed that higher doses of methadone are more likely to be responsible for rhabdomyolysis and AKI. However, these consequences occur even at lower doses. Another assumption is that prolonged unconsciousness is a necessary risk factor for rhabdomyolysis. However, there are reports of patients who are awake yet suffer rhabdomyolysis (Nanji and Douglas Filipenko 1983). In such cases, there may be other factors, like the time interval between consumption of the drug and obtaining medical care that explain the outcome (ie, the patient may have had a prolonged episode of unconsciousness before waking up and presenting to medical care).

### Limitation

In this review, most of the studies on methadone-induced rhabdomyolysis and AKI were case reports. Therefore, it is important to carry out more intervention and group studies to understand more about the causal factors and consequences of renal toxicity induced by methadone. Another limitation is that only a few reports and studies were found that addressed the direct effects of methadone in developing rhabdomyolysis and AKI. In addition, the direct effects of methadone on the kidneys, as opposed to rhabdomyolysis, were less frequently studied.

## Conclusion

Apart from its efficacy in opioid dependence and chronic pain treatments, methadone can lead to renal toxicity. Therefore, it is crucial to gain knowledge concerning the relationship between renal function and opioids to avoid adverse outcomes. Also, it should be noted that many cases of death from methadone intoxication are preventable by the proper drug prescription, good monitoring, and attention to the signs and symptoms of toxicity. Thus, education and preventative approaches are essential for maintaining the health of addicted patients receiving methadone.

## Abbreviations

AKI: acute kidney injury
US: United States
WHO: World Health Organization
CKD: chronic kidney disease
CYP: cytochrome P450
CPK: creatine phosphokinase
CK: creatine kinase
MRI: magnetic resonance imaging
CT: computed tomography
ADH: antidiuretic hormone
IV: intravenous
IDUs: intravenous drug users
SAA: serum amyloid A
ESRD: end-stage renal disease
ATN: acute tubular necrosis
CNS: central nervous system
